# Severe diaphoresis and fever during alcohol withdrawal cause hypovolemic shock: case report

**DOI:** 10.1186/s12888-021-03393-x

**Published:** 2021-08-04

**Authors:** Michitaka Funayama, Ryotaro Okochi, Shintaro Asada, Yusuke Shimizu, Shin Kurose, Taketo Takata

**Affiliations:** Department of Neuropsychiatry, Ashikaga Red Cross Hospital, 284-1, Yobe, Ashikaga-City, Tochigi, 3260843 Japan

**Keywords:** Alcohol withdrawal, Diaphoresis, Fever, Hypovolemic shock, Acute renal failure

## Abstract

**Background:**

Several fatal medical complications have been associated with alcohol withdrawal, such as seizure, cardiac arrhythmia, and takotsubo cardiomyopathy. However, there have been no reports on hypovolemic shock during alcohol withdrawal, although two physical signs of alcohol withdrawal, i.e., diaphoresis and fever, can lead to hypovolemia and its medical consequences.

**Case presentation:**

We describe a patient with alcohol use disorder who exhibited hypovolemic shock and its associated acute renal failure during alcohol withdrawal with severe diaphoresis and fever even though he had consumed almost the full amount of food he was offered. Given his excessive diaphoresis and fever that were related to alcohol withdrawal, his water intake was insufficient. Infusion with extracellular fluid resolved all these medical issues.

**Conclusions:**

The increased adrenergic activity associated with alcohol withdrawal might substantially increase a patient’s water-intake requirement through diaphoresis and fever and may cause severe hypovolemia and its associated medical complications.

## Background

A population-based register study including all patients admitted to the hospital who had been diagnosed with alcohol use disorder found that their life expectancy was 24–28 years shorter than that of the general population [1]. Causes of their premature death mainly involved alcohol-related organ damage, including the liver, cardiovascular system, and respiratory organs [2]. However, their medical complications were not limited to these conditions typical of long-term alcohol abuse. Indeed, several other critical conditions are associated with alcohol withdrawal syndrome, or delirium tremens, which occurs when alcohol is acutely withdrawn after persistent long-term intake. These conditions include seizures [3], arrhythmias [3], takotsubo cardiomyopathy [4], and hyperthermia [3], all of which add another risk of fatality for patients with alcohol use disorder. Mechanisms underlying these conditions involve a state of central nervous system arousal and increased adrenergic activity due to high NMDA (N-methyl-D-aspartate) level and low GABA (gamma aminobutyric acid) activity during alcohol withdrawal [5], for which the symptoms also include diaphoresis, hyperthermia, tremor, nausea, hallucinations or illusions, psychomotor agitation, insomnia, and anxiety [3]. In particular, both diaphoresis and fever increase the water intake requirement [6–8], which, in some cases, might lead to hypovolemia and its medical consequences [9]. To our knowledge, however, there are no previous reports pertaining to such hypovolemia-associated consequences. Here we describe a patient with alcohol withdrawal syndrome who presented with excessive diaphoresis and high fever, both of which led to hypovolemic shock and its related medical condition, i.e., acute renal failure, even though he had consumed almost all the food he was offered.

## Case presentation

A 52-year-old man with a ~ 20-year history of alcohol use disorder was admitted to our emergency room with hematemesis due to the rupture of esophageal varices and subsequent hypotension. Although he had been consuming an average of 1.5 L of wine a day, which is the equivalent of 180 g of alcohol, he was unable to control his alcohol use disorder despite repeated warnings from his doctor and his family members. His medical history included alcohol-related medical conditions, i.e., Child-Pugh B liver cirrhosis and esophageal varices, as well as unstable angina, which required coronary artery bypass grafting 7 years ago. His family members had no history of alcohol use disorder. At the emergency room, his systolic blood pressure was as low as 70 mmHg and his hemoglobin level was 7.5 g/dl (normal range for men: 13.7–16.8) (Table 1). An emergency endoscopic variceal ligation was conducted, which stopped his hematemesis. In addition, he immediately received a transfusion of 4 units of packed red cells. As a consequence, blood pressure rose to 96/63 mmHg, and he was given infusion therapy and was on nothing-by-mouth status until the fourth hospital day, when his hemostasis was confirmed by esophagogastroduodenoscopy. He was then given the standard therapeutic diet for liver disease (1500-kcal meal containing 1250 ml of water), which he consumed almost completely (Table 1). In addition, although the exact amount of his water intake was not recorded, he kept drinking water during hospitalization—at least when taking medication three times a day. His prescribed medications included aspirin, nicorandil (a vasodilator), esomeprazole (proton pump inhibitor), magnesium oxide (a laxative), and hypotensive drugs, i.e., telmisartan (an angiotensin II receptor antagonist) and bisoprolol fumarate (a beta blocker), all of which he had taken since he received coronary artery bypass grafting 7 years ago.

**Table 1:** The patient’s vital signs and laboratory data during alcohol withdrawal

		Hospital day	2	3	4	5	6	7	8	9
Alcohol withdrawal syndrome		none	none	none	mild	severe	severe	severe	mild	mild
Vital signs	Blood pressure (mmHg)	96/63	122/73	128/77	126/82	150/97	115/48	**75/48**	105/62	126/79
	Pulse rate (beats/min)	94	83	84	**119**	**118**	**101**	**115**	81	83
	Body temperature (°C)	36.7	36.4	36.3	36.6	36.6	**38.9**	**37.5**	**38.6**	**37.3**
In/Out	Oral food intake (%)	0%	0%	0%	33%	100%	90%	83%	75%	88%
	Infusion (ml)	3750	2100	1600	1100	1000	0	2500	2500	1000
	Urine output (ml)	1800	1900	2000	940	1200	NA	NA	NA	NA
	Diaphoresis	none	none	none	none	**mild**	**severe**	**severe**	none	None
Bio-chemical tests	Albumin (mg/dl: normal range 4.1–5.1)	3.2	2.8		3.1			3.2	2.9	2.8
	Total Bilirubin (mg/dl: normal range 0.4–1.5)	3.0	2.8		3.2			3.4	2.8	2.5
	Prothrombin Time (%, normal range 70–130)	69.1	84.7		78.3			70.5	83.4	71.7
	Ammonia (μmol/L: normal range < 75)	61	76		72			52		65
	Sodium (mmol/L: normal range 138–145)	140	137		133			135	139	136
	Chloride (mmol/L: normal range 101–108)	109	107		103			103	107	106
	Potassium (mmol/L: normal range 3.6–4.8)	4.1	3.9		3.6			4.2	3.8	4.0
	Glucose (mg/dl: normal range 73–109)	250	121		130			196	108	134
	Creatinine (mg/dl: normal range 0.7–1.1)	0.74	0.68		0.59			**3.43**	**1.23**	0.85
	Blood urea nitrogen (mg/dl: normal range 8–20)	**48.1**	16.1		6.9			**40.4**	**29.7**	13.2
	Creatine phosphokinase (IU/L: normal range 59–248)	56	27		34			1211	425	130
Blood count	White blood cells (per μl: normal range 3300–8600)	7300	6700		7700			17,900	15,100	8300
	Hemoglobin (g/dl: normal range 13.7–16.8)	7.5	8.9		10.4			9.5	9.4	9.2
	Platelet (× 10^4^: normal range 15.8–34.8^)^	6.0	6.4		9.5			29.7	30.0	28.4
Urine specific gravity (normal range 1.0–1.03)									1.03	

*Note*. *NA* Not assessed

### Alcohol withdrawal syndrome

The patient was fully conscious until the fourth hospital day, when he showed sinus tachycardia with a pulse rate of 119 beats/min and complained of insomnia, which required the administration of 1 mg eszopiclone, a benzodiazepine receptor agonist. The following day (the fifth hospital day), his symptoms of alcohol withdrawal syndrome worsened and presented with other typical signs, including physical signs, i.e., tremor, severe diaphoresis, and tachycardia, as well as mental signs, i.e., mildly impaired consciousness evaluated using the Glasgow Coma Scale (score of 12) (E4V3M5), visual and auditory hallucinations such as a police car and its siren sounds, illusions such as a ceiling that is moving, and agitated, restless, and violent behaviors. His delirium-associated behaviors were extremely dangerous: he attempted to break windows although a group of staff stopped him from doing so when he thought his mother was kidnapped and he had to help her. He was prescribed a total of 15 mg olanzapine (10 mg injection and 5 mg oral tablet), an antipsychotic, which effectively calmed him. In the early morning of the following day (the sixth hospital day), however, he overused his nitroglycerin oral spray without permission, which he secretly possessed for his unstable angina, when he impulsively had a suicidal ideation. The overuse of nitroglycerin caused his blood pressure to drop to 75/45 mmHg, which rose again to 115/48 in an hour without any intervention. He was then transferred to our neuropsychiatric unit, where he remained disoriented, restless, and agitated with hallucinations, and presented with tremor and substantial diaphoresis. Because he consumed almost all the meals he was offered and his behaviors were violent, we did not administer infusion therapy for fear of requiring physical restraint. Although he was deprived of his nitroglycerin, his blood pressure gradually dropped again in the afternoon, down to 86/55 mmHg in the evening. Because of his hypotension, we refrained from administering several types of psychotropic drugs, and he was prescribed only 5 mg oral olanzapine (an antipsychotic) for his alcohol withdrawal syndrome along with bisoprolol fumarate (a beta blocker), which had been prescribed for his unstable angina and is considered effective for tachycardia associated with alcohol withdrawal syndrome [10]. He, however, remained restless and kept moving around the unit without sleeping or resting. On the evening of the same day (5 p.m. on the sixth hospital day), his body temperature rose to 38.9 °C, which was 36.4 °C at 10 a.m. and 37.1 °C at 1 p.m. Oral acetaminophen (400 mg) was used to manage his fever, which dropped down to 36.9 °C in 3 hours. He revealed no remarkable physical signs of infection, such as cough, sputum, headache, fatigue, arthritis, or abdominal pain. Negative results were found for both the white blood cell count in urine and the blood culture test. His fever lasted for 4 consecutive days and resolved without antibiotic administration, as the alcohol withdrawal syndrome subsided, and thus his fever was attributed to the alcohol withdrawal syndrome itself [3].

### Hypovolemia and its associated conditions

In the morning of the following day (the seventh hospital day), his blood pressure further dropped to 75/48 mmHg with sinus tachycardia with a pulse rate of 115 beats/min while he was restlessly moving around with substantial diaphoresis. He insisted that a patient in the unit was his close relative, and he spoke with for a long time, although the person was in fact a stranger to him. His laboratory data (Table 1) showed that he had acute renal failure with a creatinine level of 3.43 mg/dl, which had been 0.59 mg/dl 3 days earlier, and a blood urea nitrogen level of 40.4 mg/dl, which was low at 6.9 mg/dl 3 days earlier. Retrospectively, the patient’s hypotension since the afternoon of the sixth hospital day could not be attributed to overuse of nitroglycerin because the half-life of nitroglycerin oral spray is only ~ 40 min (https://www.rxlist.com/nitrolingual-pumpspray-drug.htm#description). The fact that his hypotension after the overuse improved in an hour without any intervention supports this view. Also, his hypotension is not considered to be caused by hypotensive drugs, i.e., telmisartan and bisoprolol fumarate, because he had taken them for 7 years without any side effects. A gradual decline in fluid infusion after admission is unable to explain his hypovolemia because it was replaced by his oral food intake and he had consumed almost the full amount of food he was offered when his alcohol withdrawal syndrome was severe (from the fifth to seventh hospital day) and when his hypovolemic shock was observed on the seventh hospital day (Table 1). Although urine output was unable to be measured from the six hospital day due to psychiatric symptoms associated with alcohol withdrawal syndrome, the amount of his total urine output on the fifth hospital day was 1000 ml, suggesting fluid control was well managed at least until the fifth hospital day. Similarly, it is difficult to attribute his hypovolemia only to his fever because his fever on the sixth hospital day only lasted several hours before his hypovolemic shock occurred on the morning of the following day (the seventh hospital day). These data suggested that hypovolemia due to excessive diaphoresis and high fever was the mechanism underlying these medical conditions, i.e., hypotension and acute renal failure. Elevated levels of albumin and hemoglobin on the fourth and seventh hospital days were also indicative of hypovolemia, along with the urine specific gravity of 1.030, which is the upper limit of its normal range. Elevated level of white blood cell count on the seventh hospital day was most likely caused by physical stress with regard to hypovolemic shock as well as fever [11]. Delirium was considered to be a result of alcohol withdrawal syndrome because other potential factors associated with delirium were ruled out: his serum levels of ammonia and sodium remained normal, and serum glucose was above the lower limit of the normal range (Table 1), suggesting the absence of hepatic encephalopathy, hyponatremia, or hypoglycemia. Likewise, a possibility that hypovolemia and its medical consequences themselves might have caused delirium can be ruled out because his alcohol withdrawal syndrome started with insomnia and tachycardia on the fourth hospital day, when no hypovolemia or renal failure were observed (Table 1). No neurological findings were revealed except for tremor, a hallmark of alcohol withdrawal syndrome, suggesting that no brain damage had occurred, which can also cause delirium. In particular, no signs of Wernicke encephalopathy were found, e.g., ophthalmoplegia, nystagmus, or ataxia, which results from vitamin B-1 deficiency mainly due to alcohol use disorder. It was considered possible that his acute renal failure was caused by rhabdomyolysis, but this was not the case for this patient because his elevation of creatine phosphokinase was 1211 IU/L (Table 1), which is less than 10 times the upper limit of the normal range (248 IU/L × 10, or 2480 IU/L), a standard criterion for rhabdomyolysis [12]. In addition, the prompt resolution of the acute renal failure contradicts the relatively long duration of renal failure caused by rhabdomyolysis, the resolution of which takes an average of 11 days (7–17) [13].

### Infusion therapy with extracellular fluid

Considering hypovolemia as the cause underlying the patient’s medical complications, infusion therapy of extracellular fluid (2500 ml/day) was administered on the seventh day of hospitalization (Table 1). A hypotensive drug, telmisartan, was discontinued. Regarding treatment for alcohol withdrawal syndrome, 2 mg lorazepam was injected immediately, which was followed by 3 mg/day of oral lorazepam therapy for a week (from the seventh to ninth hospital day) and a reduced amount of 1.5 mg/day from the tenth hospital day. Regarding antipsychotic medications, 5 mg oral olanzapine was administered until the eighth hospital day. The patient’s hypovolemia, hypotension, and acute renal failure as well as symptoms related to alcohol withdrawal syndrome were greatly improved with these interventions, and he experienced only mild disorientation. His score on the Japanese version of the Mini-Mental State Examination (MMSE) on the thirteenth hospital day was 21 of 30, which was slightly below the cutoff score of 23/24. He became fully oriented and was discharged from our hospital on hospital day 32. On our advice, he continued to abstain from drinking and resumed his work 1 month after discharge from the hospital, when his score on the MMSE improved to 28. His clinical course, i.e., that an adequate amount of extracellular fluid infusion therapy improved all the medical conditions, certified the diagnosis of hypovolemia due to excessive diaphoresis and high fever that were related to alcohol withdrawal.

## Conclusions

This case shows that excessive diaphoresis and high fever associated with alcohol withdrawal syndrome can cause hypovolemia and its related conditions, i.e., hypotension and acute renal failure, even though a patient may consume almost all their food. Prophylactic administration of benzodiazepines immediately after admission should have been conducted and might have prevented alcohol withdrawal syndrome and these medical consequences. In our opinion, however, this clinical course might be common, considering the frequent occurrence of diaphoresis and fever during alcohol withdrawal syndrome [3], and some patients even experience iatrogenic benzodiazepine-associated delirium during alcohol withdrawal treatment [14]. In fact, diaphoresis is included as one of the essential signs for diagnosis of alcohol withdrawal syndrome in ICD-10 (International Classification of Diseases, 10th revision).

Excessive diaphoresis causes substantial water loss and can lead to fatal hypovolemic shock [6, 7, 9]. Likewise, fever increases the evaporative water loss through the skin or from the respiratory tract [15], which contributes to hypovolemia. Hypovolemia itself can increase heat storage and reduce one’s ability to tolerate heat stress [7], which poses a risk for patients with alcohol use disorder who often have several alcohol-related medical complications [2], as was true for the present case. The need for adequate hydration during alcohol withdrawal has been stated [3, 5], although this was not mentioned in the context of diaphoresis but of general care for patients with alcohol use disorder. In the clinical practice of psychiatry, however, the close relationship between diaphoresis and hypovolemia has been described in the context of neuroleptic malignant syndrome or malignant catatonia [8, 16], for which the symptoms resemble alcohol withdrawal syndrome [17]. Both conditions feature increased adrenergic activity and exhibit its related medical conditions, e.g., diaphoresis, fever, hyperthermia, and arrhythmia [3, 18].

Some limitations must be considered when interpreting the present case. Firstly, we did not record the amount of the patient’s oral fluid intake. Secondly, the amount of the patient’s perspiration was unable to be measured. Measurement of these amounts might have allowed us to precisely control fluid management. Finally, a major limitation is that this was just a single case report. Further cases should be collected to better understand water-intake requirement during alcohol withdrawal.

In conclusion, although this is just a case presentation, our report suggests that increased adrenergic activity during alcohol withdrawal might in some cases substantially increase the water requirement through excessive diaphoresis and high fever and may cause severe hypovolemia and its medical consequences. Despite severe psychiatric symptoms, meticulous care for fluid management should be taken for patients with alcohol withdrawal syndrome to reduce the risk of adverse medical consequences.

## Data Availability

All data generated or analysed during this study are included in this published article.

## Abbreviation

MMSE: Mini-Mental State Examination
